# Sindbis Virus Antibody Seroprevalence in Central Plateau Populations, South Africa

**DOI:** 10.3201/eid2810.211798

**Published:** 2022-10

**Authors:** Nicole Kennedy, Dominique Goedhals, Sabeehah Vawda, Philip Armand Bester, Felicity Burt

**Affiliations:** University of the Free State, Bloemfontein, South Africa (N. Kennedy, D. Goedhals, S. Vawda, P.A. Bester, F. Burt);; National Health Laboratory Service, Bloemfontein, South Africa (D. Goedhals, S. Vawda, P.A. Bester, F. Burt)

**Keywords:** Sindbis virus, Vector-borne infections, viruses, zoonoses, arthritis, serosurveillance, antibody, mosquitoborne, South Africa

## Abstract

We report a higher percentage of Sindbis virus-specific IgG in serum from patients attending a rheumatology clinic (18.8%) compared with healthy residents (9.6%) and patients with acute febrile illness (9.4%) in Free State Province, South Africa. Sindbis virus infection should be considered a potential cause of arthritis in South Africa.

Sindbis virus (SINV) is a mosquitoborne virus that belongs to the Togaviridae family; SINV is considered an arthritogenic alphavirus, which is known to cause self-limiting acute febrile illness (AFI) in Africa, Australia, Asia, and Europe and occasional debilitating arthritis that can persist for years after infection (*1*). Outbreaks are associated with heavy rainfall and temperature changes that favor mosquito breeding. Associations between SINV infection and acute or chronic arthralgia and myalgia have been described in Finland and Sweden (*2*,*3*). The extent of chronic debilitating disease caused by SINV in South Africa remains largely unknown.

SINV was identified as a cause of human disease in South Africa in 1963, and subsequent studies confirmed that the virus was present in mosquito populations in the central plateau region, which includes Free State Province (*4*). We investigated the seroprevalence of SINV in selected human populations of Free State Province. We used an in-house ELISA to detect SINV-specific IgG in serum and confirmed positive serum samples using neutralization assays (Appendix). We screened a total of 568 stored serum samples retrospectively and anonymously. All available stored samples were tested and included 165 serum specimens submitted to the Division of Virology, National Health Laboratory Service, for routine clinical pathology tests from patients who attended the rheumatology clinic at the Universitas Hospital, Bloemfontein, South Africa, during 2013–2017 and 267 serum samples submitted to the National Health Laboratory Service during 2008–2010 from patients with AFI and no confirmed diagnosis. No clinical data were available; however, most attendees at the rheumatology clinic had chronic arthritis. We also included 136 serum samples from healthy volunteers that were collected during 2016–2017 for seroepidemiology studies of Crimean-Congo hemorrhagic fever virus and other vectorborne diseases. 

We confirmed 11 serum samples were negative for SINV antibodies using a commercial immunofluorescence assay (EuroImmun, https://www.euroimmun.com); these samples were used to determine ELISA cutoff values. Positive control serum was obtained from 1 patient who had a laboratory-confirmed SINV infection. We obtained institutional ethics approval for this study from the Health Sciences Research Ethics Committee, University of the Free State (HSREC approval no. 95/2016C), and informed consent was available for samples collected for the seroepidemiology study (HSREC approval no. 34/2016), negative control serum panel (approval no. ETOVS 152/06), and positive control (approval no. ETOVS 118/06).

We determined optimal reagent dilutions for the ELISA using checkerboard titrations. We diluted serum samples 1:100 and tested for reactions to SINV-specific and mock antigens (Appendix). We detected reactions using horse radish peroxidase-conjugated antihuman IgG (1:8000) and 2,2′-azino-di-3-ethylbenzthiazoline-6-sulfonate (SeraCare Life Sciences, https://www.seracare.com). We measured optical density (OD) values at 405 nm and calculated net OD values by subtracting each sample OD obtained with mock antigen from the OD value obtained with SINV antigen. To normalize data, percent positivity (PP) for each sample was calculated as PP = (mean net sample OD ÷ mean net OD of the positive control) × 100.

We used the mean PP value +2 SD for 11 SINV-negative serum samples derived from a total of 83 replicates to determine the cutoff value between positive and negative samples (Appendix). We tested SINV antibody-positive serum samples for neutralizing antibodies using a 50% tissue culture infectious dose serum neutralization assay; samples were considered positive for neutralizing antibodies if the titer was >log_10_ 1.0, equal to a serum dilution >1:10 (*5*).

We detected SINV antibodies in 31/165 (18.8%) serum samples from patients who attended the rheumatology clinic, 13/136 (9.6%) samples from residents of SINV-endemic regions (high risk), and 25/267 (9.4%) samples from patients with AFI but no diagnosis (Table). Of the total number of SINV-positive samples, ≈45% of the samples were from patients who attended the rheumatology clinic (Table). We detected neutralizing antibodies with endpoint titers ranging from 1:20 to >1:640 in 65 of 69 SINV antibody-positive serum samples; 4 samples showed discordant results.

**Table:** Sindbis virus–specific IgG and neutralizing antibodies in patient serum samples in study of Sindbis virus antibody seroprevalence in central plateau populations, South Africa*

Patient group	Collection period, y	Patient samples			
		No. IgG+/no. tested per group (%)	% IgG+ of total tested	No. IgG+/total IgG+ (%)	No. NA+/no. IgG+ tested
Patients attending rheumatology clinic	2013–2017	31/165 (18.8)	5.5	31/69 (44.9)	29/31
High-risk populations†	2016–2017	13/136 (9.6)	2.3	13/69 (18.8)	13/13
Patients with AFI, no diagnosis	2007– 2010	25/267 (9.4)	4.4	25/69 (36.2)	23/25
Total no. patients		69/568	12.2		65/69

*We used an in-house ELISA to measure Sindbis virus–specific IgG and a 50% tissue culture infectious dose serum neutralization assay to measure Sindbis virus neutralizing antibodies in patient serum samples. AFI, acute febrile illness; NA, neutralizing antibody; +, positive. †Residents of Sindbis virus-endemic regions.

SINV seroprevalence in South Africa for 2006–2009 was 5.4% and increased to 12% after heavy rainfalls in 2010 (*6*). A 9.4%–9.6% seroprevalence in persons at high risk and for febrile patients is within an expected range, considering that the samples were collected over a 10-year period during which substantial rainfall in Free State Province was associated with arbovirus outbreaks (*7*,*8*). Rheumatology clinic attendees had the highest percentage (18.8%) of samples with SINV-specific IgG, compared with 9.4% for residents from SINV-endemic regions and 9.6% for patients with AFI. However, limitations exist when comparing cohorts collected at different time points, and undetected outbreaks might have been responsible for higher seroprevalence among the rheumatology clinic patients. Prospective studies in national tertiary or specialist healthcare clinics should elucidate the contribution of viral infections to chronic arthritis. Our results suggest that associations between SINV infections and arthritis have been underreported in South Africa, and SINV infection should be considered a potential cause of arthritis in this country. 

Appendix Additional information for Sindbis virus antibody seroprevalence in central plateau populations, South Africa.
