# Supplementary material for: Sindbis Virus Antibody Seroprevalence in Central Plateau Populations, South Africa
Source: Emerg Infect Dis. 2022 Oct;28(10):2137–9. doi: 10.3201/eid2810.211798 (PMC9514352; doi:10.3201/eid2810.211798)
Supplement: Appendix — Additional information for Sindbis virus antibody seroprevalence in central plateau populations, South Africa. [file 21-1798-Techapp-s1.pdf]

Article DOI: https://doi.org/10.3201/eid2810.211798

Sindbis Virus Antibody Seroprevalence in Central Plateau Populations, South Africa

Appendix

Materials and Methods

ELISA

We developed and used an in-house ELISA to screen a total of 568 patient serum samples. We prepared SINV-specific antigens from whole cell lysates of SINV-infected Vero cells. Mock antigen was prepared similarly by using uninfected cells. We tested a panel of 11 negative serum samples from volunteers and used a total of 83 replicates to determine the positive versus negative cutoff value for SINV-specific IgG antibodies. We used the mean net optical density (OD) plus 2 SD to determine a cutoff value of 0.253 (Appendix Table 1). To normalize data, we calculated percent positivity (PP) for each sample as PP = (mean net sample OD ÷ mean net OD of the positive control) x 100. The PP was 21.88%. The coefficient of variation for the ELISA was 0.21%. A coefficient of variation of <10% indicated efficient coating of the ELISA plate.

Serum samples tested positive for SINV-specific IgG antibody in 31/165 patients who attended the rheumatology clinic, 13/136 from residents of SINV-endemic regions (high risk), and 25/267 patients with acute febrile illness (AFI) but no diagnosis (Appendix Figure 1, Panels A–C). Approximately 45% of samples that tested positive for SINV-specific IgG antibodies were from patients who attended the rheumatology clinic. Using the Z-test, we found no statistical difference in IgG positivity between samples from the rheumatology clinic and population at high risk or patients with AFI ($p>0.05$). However, we observed a statistical difference in % of IgG positive samples between residents in SINV-endemic regions and patients with AFI ($p<0.05$). We observed the highest percentage of SINV-specific IgG positive samples in patients 50–59 years of age who attended the rheumatology clinic (Appendix Table 2), whereas no

specific age group had a higher percentage in the population at high risk for Sindbis virus infection (Appendix Table 3).

Neutralization Assay

We confirmed the presence of SINV-specific IgG in serum samples using a neutralization assay. We recorded titers as the reciprocal of the highest serum dilution producing a positive result. Antibody titers of ≥20 were considered positive, and an antibody titer of ≤10 was considered indeterminate. We found detectable neutralizing antibody with a titer ≥20 in 65/69 IgG-positive samples; 1/69 samples was negative, and 3/69 were indeterminate (titer ≤10) (Appendix Figure 2).

Appendix Table 1. Cut-off value for SINV-specific IgG antibodies calculated from the negative control serum panel in study of Sindbis virus antibody seroprevalence in central plateau populations, South Africa*

				Cut-off value	
Serum	No. replicates	Mean	SD	OD value	PP
Negative panel	83	0.057	0.098	0.253	21.88%

*6 Sindbis virus negative samples tested using 8 replicates each plus 5 negative samples tested using 7 replicates each. OD, optical density; PP, percent positivity.

Appendix Table 2. Demographic data and % SINV IgG antibody-positive samples of patients who attended the rheumatology clinic in study of Sindbis virus antibody seroprevalence in central plateau populations, South Africa*

	Male patients				Female patients			
Age, y†	No. patients	No. IgG+/no. patients (%)	% IgG+ of total male patients	% IgG+ of M/F patients	No. patients	No. IgG+/no. patients (%)	% IgG+ of total female patients	% IgG+ of M/F patients
0–9	1	1/1 (100)	2.6	0.6	0	0/0 (0)	0	0
10–19	4	0/4 (0)	0	0	3	0/0 (0)	0	0
20–29	3	0/3 (0)	0	0	18	2/18 (11.1)	1.6	1.2
30–39	4	0/4 (0)	0	0	23	4/23 (17.4)	3.1	2.4
40–49	5	1/5 (20)	2.6	0.6	23	2/23 (8.7)	1.6	1.2
50–59	16	6/16 (37.5)	15.7	3.6	38	9/38 (23.7)	7.1	5.5
60–69	3	1/3 (33.3)	2.6	0.6	14	2/14 (14.3)	1.6	1.2
70–79	2	1/2 (50)	2.6	0.6	5	1/5 (20)	0.8	0.6
80–89	0	0/0 (0)	0	0	3	1/3 (33.3)	0.8	0.6
Total‡	38	10/38	–	–	127	21/127	–	–

*We used an in-house ELISA to measure Sindbis virus-specific IgG antibodies in patient serum samples. SINV, Sindbis virus.
†The age range for male patients was 0.49–78 years of age and 13–81 years of age for female patients.
‡Of 165 patients, 31 tested positive for SINV-specific IgG antibodies in serum.

Appendix Table 3. Age distribution and % SINV IgG antibody positive samples of male residents in SINV-endemic regions in study of Sindbis virus antibody seroprevalence in central plateau populations, South Africa*

Age group, y†	No. patients	No. IgG+/No. patients (%)	% IgG+ of total no. patients tested
10–19	14	1/14 (7.1)	0.7
20–29	43	3/43 (7.0)	2.2
30–39	26	3/26 (11.5)	2.2
40–49	29	1/29 (3.4)	0.7
50–59	14	3/14 (21.4)	2.2
60–69	7	2/7 (28.5)	1.4
70–79	3	0/3 (0)	0
Total	136	13/136	9.6

*We used an in-house ELISA to measure Sindbis virus-specific IgG antibodies in patient serum samples; all samples were from male patients. SINV, Sindbis virus.
†The age range for male patients was 18–76 years of age.

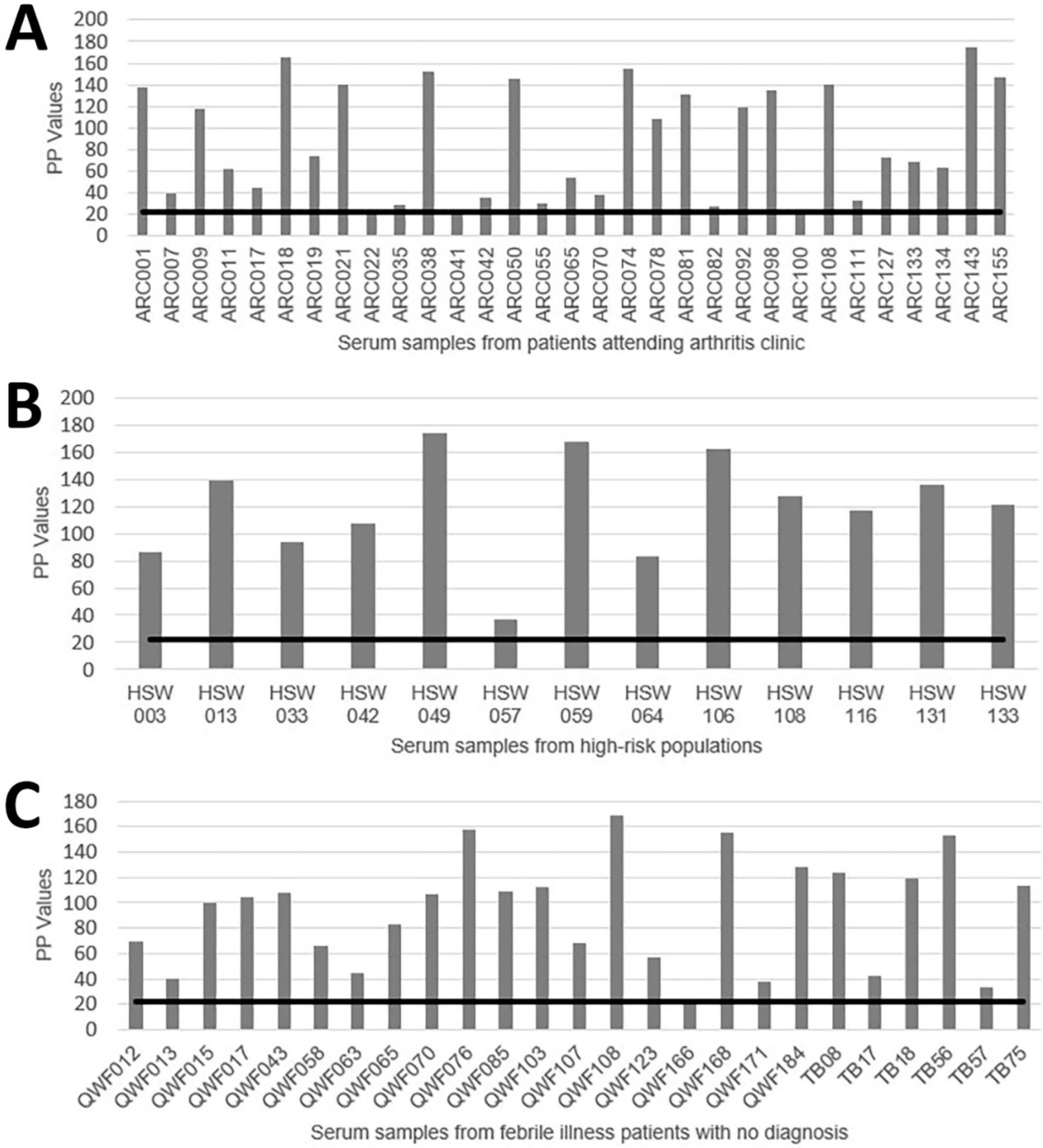

Appendix Figure 1. Percent positivity values for SINV-specific IgG antibodies in patient serum samples in a study of Sindbis virus antibody seroprevalence in central plateau populations, South Africa. The PP for each sample was calculated as PP = (mean net sample OD ÷ mean net OD of the positive control) x 100. We calculated a cutoff value of 21.88% that differentiated antibody-positive from antibody-negative samples. A) PP values for patients who attended the arthritis clinic (ARC), B) PP values for residents of SINV-endemic regions with high risk for infection (HSW), and C) PP values for patients with febrile illness (QWF). OD, optical density; PP, percent positivity; SINV, Sindbis virus.

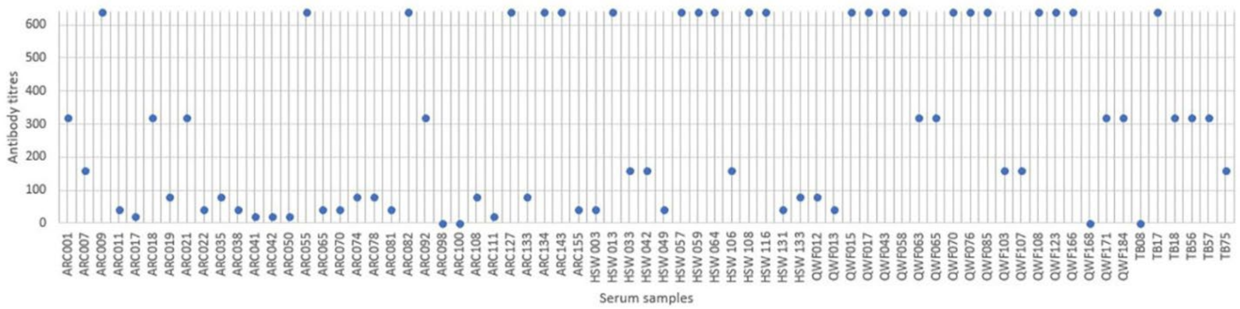

Appendix Figure 2. Antibody titers for 69 SINV-specific IgG-positive serum samples that were tested by using the neutralization assay in a study of Sindbis virus antibody seroprevalence in central plateau populations, South Africa. We recorded titers as the reciprocal of the highest serum dilution producing a positive result. Antibody titers of $\geq$20 were considered positive, and an antibody titer of $\leq$10 was considered indeterminate.
